# Structural Insight on Functional Regulation of Human MINERVA Protein

**DOI:** 10.3390/ijms21218186

**Published:** 2020-10-31

**Authors:** Hyunggu Hahn, Dong-Eun Lee, Dong Man Jang, Jiyoun Kim, Yeon Lee, Heesun Cheong, Byung Woo Han, Hyoun Sook Kim

**Affiliations:** 1 Research Institute, National Cancer Center, Goyang, Gyeonggi 10408, Korea; hahnhk@ncc.re.kr (H.H.); de74719@ncc.re.kr (D.-E.L.); zhijuan325@naver.com (J.K.); ylee@ncc.re.kr (Y.L.); 2 Research Institute of Pharmaceutical Sciences, College of Pharmacy, Seoul National University, Seoul 08826, Korea; jdm721@snu.ac.kr; 3 Graduate School of Cancer Science & Policy, National Cancer Center, Goyang, Gyeonggi 10408, Korea

**Keywords:** MINERVA, FAM129B, PH domain, lipid binding, novel fold, cancer progression, invasion

## Abstract

MINERVA (melanoma invasion by ERK), also known as FAM129B, is a member of the FAM129 protein family, which is only present in vertebrates. MINERVA is involved in key signaling pathways regulating cell survival, proliferation and apoptosis and found upregulated in many types of cancer promoting invasion. However, the exact function of the protein remains elusive. X-ray crystallographic methods were implemented to determine the crystal structure of MINERVA^ΔC^, lacking C-terminal flexible region. Trypsin digestion was required before crystallization to obtain diffraction-quality crystals. While the N-terminal pleckstrin homology (PH) domain exhibits the typical fold of PH domains, lipid binding assay indicates specific affinity towards phosphatidic acid and inositol 3-phosphate. A helix-rich domain that constitutes the rest of the molecule demonstrates a novel L-shaped fold that encompasses the PH domain. The overall structure of MINERVA^ΔC^ with binding assays and cell-based experiments suggest plasma membrane association of MINERVA and its function seem to be tightly regulated by various motifs within the C-terminal flexible region. Elucidation of MINERVA^ΔC^ structure presents a novel fold for an α-helix bundle domain that would provide a binding platform for interacting partners.

## 1. Introduction

In human proteome, the pleckstrin homology (PH) domain is present in nearly 300 proteins involved in diverse physiological processes. A typical PH domain comprises about 100 amino acid residues structured into a seven-stranded antiparallel β-sheet and a C-terminal α-helix [1]. The most well-known physiological property of PH domains is recognition of phosphoinositides with high affinity and specificity, which regulate localization of PH domain-containing proteins to and from intracellular membranes [2]. PH domains recognizing phosphoinositide display polar surface charge distribution around its β1–β2 region for accommodation of negatively charged head groups of phosphoinositides. On the other hand, PH domains also contribute to binding interaction partner proteins, including G-protein coupled receptors (GPCRs), GTPase exchange factors (GEFs), and focal adhesion kinase (FAK) [3,4]. Phosphotyrosine-containing peptides and polyproline helixes may bind on pockets created by the β-sheet and the C-terminal α-helix of the PH domain [5]. Considering its pivotal role in regulating protein subcellular localization, mutations within the PH domains can lead to aberrant cell signaling [4].

FAM129B (family with sequence similarity 129, member B), also known as MINERVA (melanoma invasion by ERK), is a member of a small family of proteins that includes FAM129A and FAM129C, which are present only in vertebrates. Sequence analysis of the FAM129 family members reveals that they share a common PH domain on their N-terminal region with high sequence similarity of 57% between MINERVA and FAM129A, and 40% between MINERVA and FAM129C [6], even though the roles of the PH domains in their functions are not well understood. While FAM129A is an endoplasmic reticulum stress-induced protein that is upregulated in renal and thyroid cancer [7], FAM129C is a B-cell membrane protein that is overexpressed in chronic lymphocytic leukemia [8].

Following the first identification of MINERVA as a target of ERK phosphorylation [6], several studies have reported that MINERVA is upregulated in many types of cancer, including breast, kidney, large intestine, lung and endometrial cancers as well as hematopoietic and central nervous system tumors [9,10]. Moreover, cancer patients overexpressing MINERVA exhibited bad prognosis and low survival rate [11,12]. Overexpression of MINERVA was associated with activation of focal adhesion kinase (FAK) by facilitating phosphorylation of Tyr397 and Tyr925 residues on FAK [11], which is associated with increased cell migration [13]. Similar observations were made from MINERVA-deficient mice that exhibited a delayed rate of wound healing, suggesting a critical role of MINERVA in promoting cell motility [14].

MINERVA is a target of phosphorylation by both epidermal growth factor receptor (EGFR) and ERK, the central kinases in the ERK growth signaling pathway [6,15]. EGFR is a receptor tyrosine kinase that phosphorylates MINERVA on its Tyr593 residue in response to extracellular growth signaling molecule epidermal growth factor (EGF). MINERVA phosphorylated on Tyr593 is capable of direct binding to H-Ras and K-Ras for their constitutive activation by reducing association of Ras GTPase-activating proteins (GAPs) to inhibit Ras-bound GTP hydrolysis [15]. Constitutive activation of Ras proteins in turn would activate downstream ERK for β-catenin-TCF/LEF transcriptional complex-mediated upregulation of cyclin D1, c-Myc, and PKM2 for promotion of Warburg effect, thereby promoting cell proliferation and tumorigenesis [15]. ERK also phosphorylates multiple serine residues on the C-terminal flexible region of MINERVA as mentioned above, enhancing invasion capabilities of cells presumably via the activation of FAK [6,11]. In sparse, exponentially growing HeLa cells, MINERVA is localized in cytoplasm, whereas the protein is translocalized into plasma membrane colocalizing with β-catenin at adherence junctions in growth-inhibited confluent cells [16]. The localization of MINERVA at the adherence junctions seems to protect β-catenin from proteolytic degradation, delaying onset of apoptosis elicited by the cellular confluency [16]. Moreover, MINERVA interacts with LATS1 kinase that suppresses nuclear transportation of YAP, suggesting its involvement in the Hippo signaling pathway activated in confluency [17]. Overall, MINERVA modulates crosstalk among essential cellular signaling pathways for cell proliferation, invasion, and apoptosis.

Sequence and secondary structure analyses of 746 amino acid-long MINERVA protein indicate a PH domain on the N-terminus (residues Asp66–Gly197) and a proline-rich region on the C-terminus (residues Asp575–Phe746) which contains the phosphorylatable serine residues [6]. PH domains are well-known for binding phosphatidylinositol phosphates (PIPs) for regulating protein localization [2]. Additionally, MINERVA contains an N-terminal myristoylation motif ^1^-MGX_3_S-^6^ [18], which supports intracellular membrane localization of MINERVA [16]. Within the C-terminal region, there is a ^708^-DLGX_7_ETGE-^721^ motif that interacts with Kelch domain on Kelch-like ECH-associated protein 1 (Keap1), which is involved in Keap1-mediated ubiquitination of nuclear factor-erythroid 2-related factor 2 (Nrf2) for proteasomal degradation [12]. Nrf2 is a key regulator in cellular response against oxidative stress by regulating transcription of antioxidant gene expression that is targeted for degradation under quiescent conditions [19,20]. Hence, MINERVA was suggested to act as an upstream regulator of Nrf2-mediated response to oxidative stress in cancer by hindering interaction of Keap1 and Nrf2 by competing for the Kelch domain through the “DLGX_n_ETGE” motif [12].

Despite the importance of MINERVA in regulating cell growth, migration, apoptosis, and oxidative stress, no structural information of the protein or other FAM129 family members was available. Here, we report the first crystal structure of MINERVA lacking the C-terminal flexible region, which contains the PH domain and a helix bundle domain with a novel fold. In addition, lipid binding ability of the PH domain is assessed for structural and functional analyses of MINERVA. This study shows structural basis for membrane association of MINERVA and its importance in governing multiple signaling pathways in cancer progression.

## 2. Results

### 2.1. Functional Analyses of Physiological Role of MINERVA

**Figure 1:**
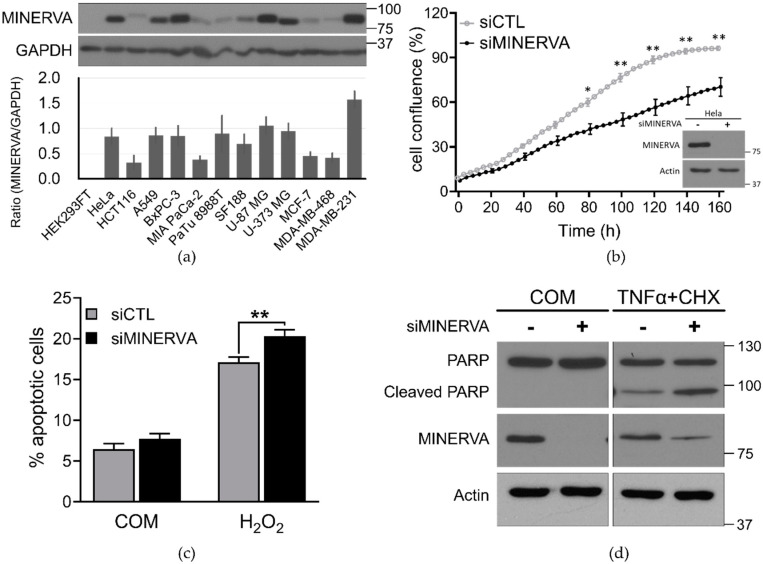
Higher expressions of melanoma invasion by ERK (MINERVA) in cancer cell lines. (**a**) Various cancer cell lines from multiple tumor origins; HEK293FT, HeLa (cervical cancer), HCT116 (colon cancer), A549 (lung cancer), BxPC-3, MIA PaCa-2, PaTu 8988T (pancreatic cancers), SF188, U-87 MG, U-373 MG (brain cancers), MCF-7, MDA-MB-468, and MDA-MB-231 (breast cancers) were cultured in optimal media and cell lysates were prepared individually, then immunoblotted against MINERVA. GAPDH was used as a loading control. Immunoblotted proteins from experiments performed in triplicate were quantified using Image J software (NIH, Bethesda, MD, USA). Full blot can be found in Figure S4. (**b**) HeLa cells reverse-transfected with either siRNA against MINERVA (siMINERVA) or scrambled siRNA (siCTL) were seeded into a 96-well plate. Images acquired from the IncuCyte ZOOM™ system at the indicated times were analyzed. Cell confluence was measured in triplicate wells for each time point. Error bars indicate the mean ± SEM for three independent experiments. * *p*  <  0.05, ** *p*  <  0.01. Protein extracts from siRNA-transfected cells in 72 h were analyzed for the MINERVA knockdown in western blot. Full blot can be found in Figure S5. (**c**) HeLa cells reverse-transfected with siRNA against MINERVA were incubated in culture medium with either H_2_O_2_ or vehicle for 4 h. Cell death was assessed using Annexin V/propidium iodide (PI) staining by flow cytometry. The dead cell portion was measured based on the number of annexin V and PI single-stained cells. Error bars indicate the mean ± SEM for three independent experiments. ** *p*  <  0.01. (**d**) Proteins extracted from siRNA-transfected cells were analyzed by western blotting using anti-PARP1 and anti-β-actin antibodies. The proform of PARP (116 kDa) and cleaved PARP (85 kDa) are indicated. Full blot can be found in Figure S6.

Previous studies have shown that high levels of MINERVA expression were related to increased tumor cell invasion, delayed onset of apoptosis, and ultimately low survival rate of patients with a variety of cancer types [11,12,16]. The overall survival rate of breast, brain, and pancreatic cancer patients plotted against MINERVA expression level through Kaplan–Meier analysis from The Cancer Genome Atlas datasets (TCGA 2012 provisional) [21] shows the negative effect of high MINERVA expression in cancer prognosis (Figure S1). Although it is known that MINERVA is overexpressed in various cancer cell types, the expression level was verified in a limited number of cell lines (HeLa, U251, LN229, PC14, HaCaT, or patient-derived non-small cell lung cancer (NSCLC) cells) [11,14,15,16]. To obtain more comprehensive data on expression levels of MINERVA, cancer cell lines from different tissue origins were tested. Protein expression levels of MINERVA relative to GAPDH in the cancer cells were significantly elevated compared to that of the human embryonic kidney (HEK293FT) cells used as a negative control (Figure 1a). The expression data suggest a ubiquitous role of MINERVA in cancer progression.

Next, we verified changes in cell growth and apoptosis rate in MINERVA knockdown cells. We observed that the cell growth rate of the HeLa cells treated with MINERVA siRNA was significantly reduced compared with cells treated with scrambled siRNA (siCTL) in IncuCyte ZOOM™ system (Figure 1b). In addition, apoptosis rate in response to hydrogen peroxide (H_2_O_2_) was markedly increased compared to that in siCTL-treated cells as analyzed by Annexin V and propidium iodide (PI) staining (Figure 1c). We also observed more PARP cleavage in response to TNFα treatment in siMINERVA-treated cells, compared to those treated with siCTL (Figure 1d), which is consistent with the previous observations by Chen et al. [16]. These data indicate that MINERVA plays a critical role in cancer cell proliferation and survival, especially under oxidative stress condition.

### 2.2. MINERVA Crystallization and Structure Determination

**Figure 2:**
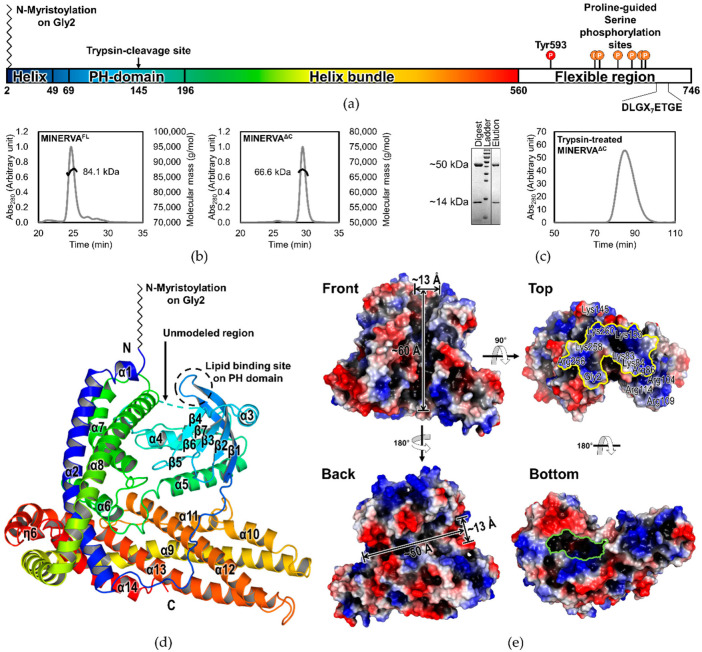
Overall structure of the human MINERVA^ΔC^. (**a**) Schematic of MINERVA sequence showing domain composition and trypsin-cleavage site, posttranslational modification sites, as well as the Keap1-binding motif ^708^-DLGX_7_ETGE-^721^. The sites of phosphorylation by epidermal growth factor receptor (EGFR) (Tyr593) and by ERK (Ser641, Ser646, Ser665, Ser681, Ser692, and Ser696) are marked with red and orange circles, respectively. The structured domain (residues Gly2–Arg560) is colored in rainbow spectrum from N-terminus to C-terminus. (**b**) Size exclusion chromatography–multiangle light scattering (SEC–MALS) results of MINERVA^FL^ and MINERVA^ΔC^ show monomeric states of the proteins. (**c**) SEC and SDS-PAGE results of trypsin-treated MINERVA^ΔC^ show that the protein after trypsin-cleavage is divided into two fragments but retains its integrity in solution. (**d**) The overall MINERVA^ΔC^ structure in cartoon representation colored in the same rainbow spectrum scheme as in (**a**). Secondary structure elements and the N- and C-termini are labeled. Unmodeled region, N-myristoylation and lipid binding site on the pleckstrin homology (PH) domain are indicated. (**e**) Surface representations of the MINERVA^ΔC^ structure showing surface electrostatic potentials. Front view is in the same orientation as MINERVA^ΔC^ in (**d**). Top view shows a surface composed of positively charged residues for membrane association (yellow outline). Front and back views show two distinct crevices traversing the whole molecule that are approximately 13 Å wide and 50 Å or 60 Å long each. Bottom view shows an entrance to a large cavity inside the molecule (green outline).

The sequence analysis result of MINERVA using various web servers such as XtalPred, PSIPRED, and DISOPRED [22,23,24] predicted that the C-terminal region of MINERVA (Asp575–Phe746) is disordered, while the N-terminal PH domain and its flanking helix-rich regions are well-conserved among the members of the FAM129 family (Figure S2). The domain composition and the known posttranslational modification (PTM) sites of MINERVA are denoted in Figure 2a. We successfully purified MINERVA^FL^ and MINERVA^ΔC^ (Figure 2b) and attempted crystallization of the proteins. Unfortunately, not only MINERVA^FL^ but also MINERVA^ΔC^ produced poorly diffracting rosette-shaped crystals, which were not fit for data collection. We suspected that internal disordered regions were hindering crystal formation and implemented limited proteolysis by trypsin. The limited trypsin treatment effectively digested MINERVA^ΔC^ and yielded two separate fragments with molecular weights of approximately 14 kDa and 50 kDa, as confirmed by SDS-PAGE (Figure 2c, left). N-terminal sequencing identified the first five residues of the fragments as Gly^2^-Asp^3^-Val^4^-Leu^5^-Ser^6^ and Ser^146^-Gly^147^-Ser^148^-Ala^149^-Pro^150^, respectively. The sequencing results corresponded with the prediction from PeptideCutter [25], that trypsin would cleave the protein after Lys145 residue. Other predicted trypsin cleavage sites were likely protected by protein folding, which prevented complete fragmentation of the protein. The trypsin-digested MINERVA^ΔC^ fragments co-eluted from the size-exclusion chromatography column (Figure 2c), meaning that the two fragments remained intact due to extensive interaction between them and the trypsin-digestion did not disrupt the overall folding of MINERVA^ΔC^. Trypsin digestion of MINERVA^FL^ also gave fragments of the same sizes (data not shown), justifying our MINERVA^ΔC^ construct design of the C-terminal flexible region removal. The trypsin digestion greatly aided production of large single crystals of MINERVA^ΔC^ suitable for data collection, and hence was implemented for crystallizations of the native and SeMet-substituted MINERVA^ΔC^.

We collected X-ray diffraction data from crystals of the trypsin-treated native and SeMet-substituted MINERVA^ΔC^ at 1.65 Å and 2.39 Å resolutions, respectively. Due to the absence of known similar structures, the phase was obtained from the SeMet-substituted MINERVA^ΔC^ crystal by the single-wavelength anomalous diffraction (SAD) method. Using the SeMet-substituted structure as a molecular replacement (MR) model, we could successfully resolve the native MINERVA^ΔC^ structure for residues Gly2–Lys145 and Leu152–Arg560 at higher resolution (Table 1). The C-terminal region as well as Ser146–Ile151 could not be modeled due to high flexibility in these regions. As the last residue on the carboxy-terminal of the model is Arg560, the missing region Lys561–Ser574 was likely cleaved by trypsin. In an asymmetric unit (ASU) of *P* 2_1_2_1_2_1_ space group, there was only one MINERVA^ΔC^. According to proteins, interfaces, surfaces, and assemblies (PISA) analysis [26], the largest surface area a MINERVA^ΔC^ molecule shares with an adjacent one is 573.2 Å^2^, suggesting that MINERVA^ΔC^ exists as a monomer. Oligomeric states of MINERVA^FL^ and MINERVA^ΔC^ in solution were assessed by measuring molecular mass using size exclusion chromatography–multiangle light scattering (SEC–MALS) method, supporting that both constructs of MINERVA exist as monomers in solution as seen in the crystal (Figure 2b).

### 2.3. Overall Structure of MINERVA^ΔC^

The overall structure model of a MINERVA^ΔC^ monomer assumes a novel globular fold that spans approximately 70 Å by 70 Å by 60 Å in size. The structure of MINERVA^ΔC^ could be divided into two domains, the PH domain (residues Ile69–Asn196) and a helix bundle domain comprising the rest of the molecule (Figure 2a), which exhibits a distorted L-shape where the PH domain is held at the corner of the helix bundle (Figure 2d). The spatial arrangement of the two domains allows the N-terminus of the protein to be located adjacent to the PH domain in proximity, presumably for cooperative action of myristoylated Gly2 residue and the PH domain for membrane localization of MINERVA (Figure 2d). Surface representation of the protein reveals that MINERVA possesses a positively charged patch on its membrane-interacting face, which is mainly composed of positively charged residues Lys83, Lys84, Arg86, Lys104, Arg109, Arg114, Lys145, and Lys153 of the PH domain and Arg256, Lys258, and Lys260 of the helix bundle domain, presumably enabling stable interactions of MINERVA with the plasma membrane (Figure 2e, top view). To our surprise, the surface representation revealed two distinct and characteristic crevices that are approximately 13Å wide and 50 Å or 60 Å long each on the front and the back, respectively (Figure 2e, front and back views), and a large cavity inside the molecule (Figure 2e, bottom view), which might serve as binding platforms for interaction partners.

The helix bundle domain is composed of 11 α-helices (α1–α2 and α6–α14) and six 3_10_ helices (η1–η6) that are held together mostly by hydrophobic contacts. The overall architecture of the helix bundle domain resembles no other known structure, as analyzed by Dali structural similarity search algorithm [27], suggesting that it is of a novel fold. Sequence alignment of the three FAM129 family members shows that this specific fold, including the N-terminal PH domain, is well-conserved among the family members (Figure S2). Interestingly, the helices α8–η2–α9 spanning 119 residues (Pro261–Glu379) are severely bent around the helix η2 (Gln340–Phe342) to encircle the entire protein, forming the characteristic L-shape (Figure 2d, green-to-yellow). Other helices in the bundle interact with the helices α8–α9 via hydrophobic contacts. Helices α2 and α7 are associated with the helix α8, while helices α10 through α14 interact with the helix α9 (Figure 2d). The N-terminal PH domain is tightly held by the helix bundle domain that acts as a pincer-like scaffold, with a large contact area of 1573.4 Å^2^. Residues on helices α6, 7, 9, and 10 comprise an extensive network of hydrogen bonds and salt bridges with residues on the PH domain, such as Glu67 and His94, and Gln434, Arg192, Gln431, Asn195, and Gly231.

**Figure 3:**
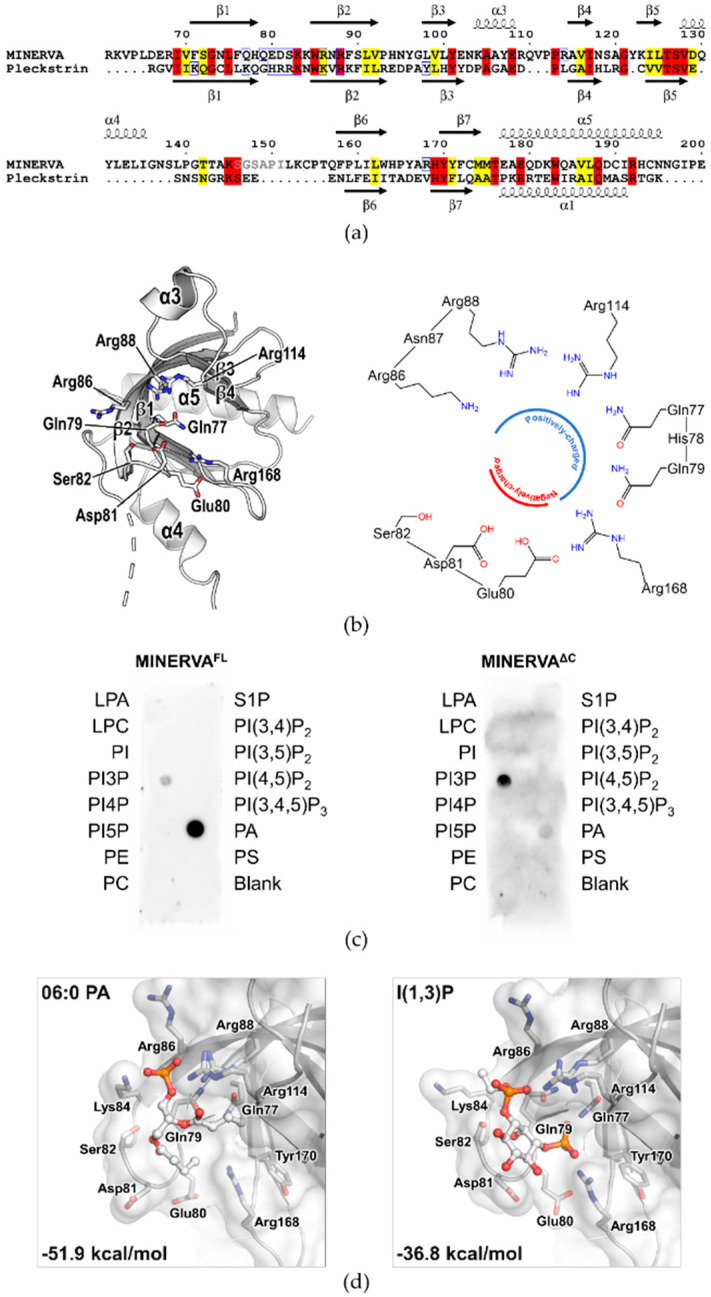
The PH domain of MINERVA. (**a**) Structure-based sequence alignment of PH domains from MINERVA and Pleckstrin (PDB ID: 2I5F) showing conserved residues at the ligand binding site. Conserved residues and similar residues are highlighted in red and yellow, respectively. Residues contributing to ligand recognition are indicated with blue frames. Secondary structure elements of the MINERVA and Pleckstrin models are shown above and below the alignment, respectively. Unmodeled residues (Ser146–Ile151) of MINERVA due to lack of electron density around the cleavage site are in gray. (**b**) Left, cartoon representation of the PH domain of MINERVA with side chains of putative ligand interacting amino acids shown in stick representation. There are both positively charged and negatively charged residues in the ligand binding pocket. Right, schematic of the residues at ligand binding pocket of MINERVA PH domain showing positively charged and negatively charged faces. (**c**) Lipid dot-blot assay of MINERVA^FL^ (left) and MINERVA^ΔC^ (right). Both MINERVAL^FL^ and MINERVA^ΔC^ are capable of recognizing PA and PI3P, but to different extents. Dotted lipids are as follows: lysophosphatidic acid (LPA), lysophosphatidylcholine (LPC), phosphatidylinositol (PI), phosphatidylinositol (3)-phosphate (PI3P), phosphatidylinositol (4)-phosphate (PI4P), phosphatidylinositol (5)-phosphate (PI5P), phosphatidylethanolamine (PE), phosphatidylcholine (PC), sphingosine 1-phosphate (S1P), phosphatidylinositol (3,4)-bisphosphate (PI(3,4)P_2_), phosphatidylinositol (3,5)-bisphosphate (PI(3,5)P_2_), phosphatidylinositol (4,5)-bisphosphate (PI(4,5)P_2_), phosphatidylinositol (3,4,5)-trisphosphate (PI(3,4,5)P_3_), phosphatidic acid (PA), and phosphatidylserine (PS). (**d**) Glide induced fit docking results of the head groups of PA (left) and PI3P (right) against the PH domain of MINERVA. Sidechains of residues for lipid binding are indicated in stick and surface representations. Binding free energy for PA and PI3P headgroups calculated by the molecular mechanics–generalized Born surface area (MMGBSA) method is shown, respectively.

The N-terminal PH domain adopts a typical fold of a PH domain containing a central curved antiparallel β-sheet (β5↑-β6↓-β7↑-β1↓-β2↑-β3↓-β4↑) followed by a C-terminal α-helix, α5 (Figure 2d). Sequence alignment of MINERVA PH domain with a representative PH domain from Pleckstrin (PDB ID: 2I5F) [28] showed moderate sequence identity and similarity of 22% and 35%, respectively. Moreover, structure-based sequence alignment of the PH domains of MINERVA and Pleckstrin revealed that the core secondary structures including the characteristic antiparallel β-sheet are well conserved (Figure 3a) with root-mean-square deviation (RMSD) of 1.30 Å over 91 equivalent C_α_ positions. On the other hand, there are two additional α-helices α3 and α4 inserted between β3 and β4, and β5 and β6, respectively, in the MINERVA PH domain (Figure 3a,b, left). A major difference between the PH domains of MINERVA and Pleckstrin arises from a loop region connecting the helix α4 and the strand β6 (residues Gly136–Phe158) of MINERVA, which is unusually long and disordered (Figure 3a). Residues Ser146–Pro151 within this region could not be modeled in our structure due to lack of electron densities. The corresponding region in Pleckstrin (residues Asn303–Glu311) was shorter but also disordered. Incidentally, the trypsin cleavage site (after Lys145) is located within the α4–β6 loop, meaning that this loop is intrinsically disordered and exposed to solvation.

### 2.4. PH Domain of MINERVA Recognizes Phosphatidic Acid and Phosphatidylinositol 3-Phosphate

PH domains are well-known for their ability to recognize phosphatidylinositols [2]. Structure-based sequence alignment of the PH domains from MINERVA and Pleckstrin in complex with d-myo-inositol 1,2,3,5,6-pentakisphosphate (IP_5_) (Figure 3a) suggests that putative binding site for lipids on MINERVA PH domain is composed of residues Gln77, Gln79, Lys84, Arg86, Arg88, Arg114, and Arg168, which are mainly located on strands β1 and β2, forming a positively charged face of its ligand binding pocket (Figure 3b). On Pleckstrin, residues Lys253, His256, Arg257, Arg258, Lys262, Arg264, and Tyr277 establish grossly positively charged surface that form extensive hydrogen bond network with the bound IP_5_. Among these residues, His256–Arg258 seemed to be the major contributors in stabilizing the bound IP_5_ by encapsulating the ligand with their long and positively charged sidechains. MINERVA possesses negatively charged amino acid residues (Glu80–Asp81–Ser82) instead on the positions corresponding to His256–Arg258 of Pleckstrin (Figure 3a), which form a negatively charged face within the ligand binding site (Figure 3b, right). The oppositely charged faces within the ligand binding site would restrict types of ligands that can bind in the MINERVA PH domain to those with fewer phosphates at their head groups. Furthermore, efficient IP_5_ binding on Pleckstrin is facilitated by an absence of side chain on Gly255 residue, while MINERVA possesses Gln79 on the corresponding position (Figure 3b, left and Figure S3a), which would cause steric hindrance to incoming ligand.

Since MINERVA is recruited to plasma membrane under certain growth-inhibited conditions [6], affinity towards the membrane lipids of the PH domains was expected. In fact, phosphatidylinositol (3)-phosphate (PI3P) was recognized as the innate ligand of the MINERVA PH domain [29]. We implemented lipid dot blot assay using PIP Strips™ to determine which lipids are recognized by MINERVA^FL^ and MINERVA^ΔC^. The dot blot assay revealed that both MINERVA^FL^ and MINERVA^ΔC^ recognized phosphatidic acid (PA) and PI3P, but to different extents (Figure 3c). MINERVA^FL^ showed significantly higher affinity towards PA over PI3P, while MINERVA^ΔC^ preferred PI3P. As shown in Figure 3b, we expected the lipid binding pocket of MINERVA to possess specific affinity towards lipids with few phosphates at their head groups due to oppositely charged faces within the ligand binding site and the steric hindrance elicited by side chain of the Gln79. PA and PI3P both satisfy these criteria, though it is not clear how other similar lipids such as PI4P and PI5P are discriminated against. Nevertheless, the lipid dot blot assay results suggest that the C-terminal flexible region (residues Asp575–Phe746) could play a role in switching specificity of the N-terminal PH domain between PA and PI3P.

Next, we attempted to solve the structure of MINERVA^ΔC^ in complex with PA or PI3P to elucidate the mechanism of ligand recognition. However, we could only obtain structures of ligand-unbound forms of MINERVA^ΔC^. We instead performed computational Glide-induced fit docking [30] of the head groups of PA or PI3P against the MINERVA PH domain to predict their binding modes and calculated binding free energy (ΔG^MM-GBSA^). As expected, both PA and PI3P were modeled well at the ligand binding site of the PH domain with binding free energies of −51.9 kcal mol^−1^ and −36.8 kcal mol^−1^, respectively, suggesting more stable binding of PA with the MINERVA PH domain. The docking results showed that the phosphate groups on PA and PI3P both establish multiple polar interactions with the positively charged residues (Lys84, Arg86, Arg88, Arg114, and Arg168) at the ligand binding site (Figure 3d). Interestingly, the negatively charged residues Glu80–Asp81 are flipped away from the ligand binding site to better accommodate the bound ligands in this model.

### 2.5. Functional Analyses of N- and C-Terminal Regions of MINERVA^FL^

**Figure 4:**
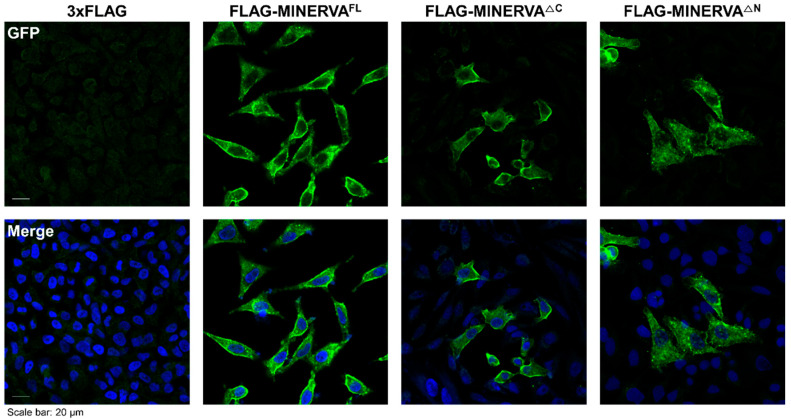
Sub-cellular localization of truncated MINERVA. After incubating HeLa cells for overnight, FLAG (DYKDDDDK)-tagged MINERVA^FL^, MINERVA^ΔC^, and MINERVA^ΔN^ were transfected into cells, respectively, by standard manufacturer’s protocol and further incubated for 48 h. Cells were fixed and stained with a Hoechst 33342 for DNA and with antibody against FLAG-epitopes for immunofluorescence analysis. The secondary antibody used was goat anti-rabbit IgG Alexa Fluor 488. Subsequently, the subcellular localizations of MINERVA constructs were monitored by confocal fluorescence microscopy. The data are representative images from 10 different images at least three independent experiments. Scale bar: 20 μm.

In the above sections, we showed by solving protein structure that MINERVA possesses an N-terminal PH domain, and that it recognizes PA and PI3P. To assess the physiological significance of the PH domain, we generated an additional N-terminal truncation construct (MINERVA^ΔN^; residues Glue307–Phe746) and compared its subcellular localization in HeLa cell along with MINERVA^FL^ and MINERVA^ΔC^. The MINERVA constructs were FLAG (DYKDDDDK)-tagged for detection and their localizations were monitored by immunofluorescence analysis. In accordance with the previous observations by Chen et al. [16], MINERVA was localized at plasma membrane. Interestingly, both MINERVA constructs retaining the N-terminal PH domain (MINERVA^FL^ and MINERVA^ΔC^) were mostly localized at plasma membrane, whereas MINERVA^ΔN^ was localized in cytoplasm as either diffused or puncta forms (Figure 4). The immunofluorescence data emphasizes the importance of the PH domain on MINERVA for membrane localization.

**Figure 5:**
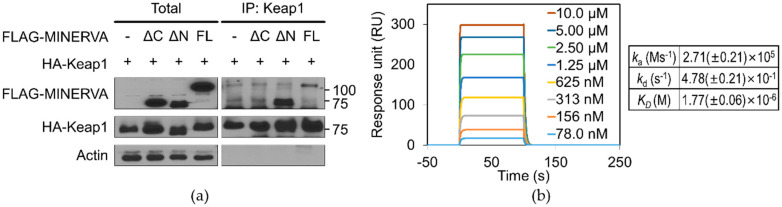
Interaction of MINERVA with Keap1 through its C-terminal region. (**a**) HEK293 cells were co-transfected with HA-tagged Keap1 and either one of the FLAG-tagged MINERVA^FL^, MINERVA^ΔN^, or MINERVA^ΔC^. HA-Keap1 was immunoprecipitated with anti-HA with protein A-Sepharose and the immunoprecipitates were examined by western blots using respective antibodies. Actin was used as a loading control. Full blot can be found in Figure S7. (**b**) Surface plasmone resonance (SPR) analysis of MINERVA^FL^ and Keap1 showing micromolar binding affinity between two proteins. The average and standard deviations of *k_a_*, *k_d_*, and *K_D_* are calculated from values obtained from two separate experiments.

Although the C-terminal flexible region was excluded from structural study due to its high flexibility, this region has been a focus of attention for functional regulation of MINERVA by multiple reports [6,12,15]. We examined interaction between MINERVA and Keap1, by co-transfecting each of FLAG-tagged MINERVA constructs (MINERVA^FL^, MINERVA^ΔN^, and MINERVA^ΔC^) with HA-tagged Keap1 and subsequently analyzing co-immunoprecipitation (co-IP). MINERVA^FL^ and MINERVA^ΔN^ were co-immunoprecipitated with Keap1, whereas MINERVA^ΔC^, which is missing the Keap1-interacting motif “DLGX_n_ETGE” located in the C-terminal flexible region [12], failed to interact with Keap1 (Figure 5a). Following the co-IP experiment, we performed surface plasmone resonance (SPR) analysis between the two proteins, with MINERVA^FL^ as ligand and Keap1 as analyte. Keap1 showed interaction with MINERVA^FL^ in a concentration dependent manner, and a micromolar *K_D_* was obtained from kinetics analysis (Figure 5b).

## 3. Discussion

Here, we report the first crystal structure of human MINERVA^ΔC^, a protein found upregulated in many types of cancer with an unclear function. Sequence analysis predicted intrinsically disordered C-terminal region containing multiple phosphorylation sites and a Keap1-interaction motif, which was excluded in our construct for structure determination. The structure of MINERVA^ΔC^ comprises the N-terminal PH domain and an α-helix bundle domain (residues Gly2–Glu49 and Glu205–Arg560; Figure 2a,d) of a novel fold. Flexible α3–β6 loop within the PH domain was cleaved by trypsin for generation of diffraction-quality crystals. To our surprise, intermolecular interaction analysis by the PISA program suggested that the cleavage allowed better stacking of the MINERVA^ΔC^ molecules in crystal lattice. Helix α8′ (residues Ser292′–Arg306′) from an adjacent MINERVA^ΔC^ molecule approaches the helix α4 region exposed by the trypsin cleavage, forming several intermolecular interactions with residues Asp129–Asn137 and Lys260–Gln265 (Figure S3b, gray dashed ribbon). These residues forming the interactions would have been concealed by the α3–β6 loop before trypsin treatment, explaining poor diffraction quality from crystals of untreated MINERVA^ΔC^ we had obtained.

**Figure 6:**
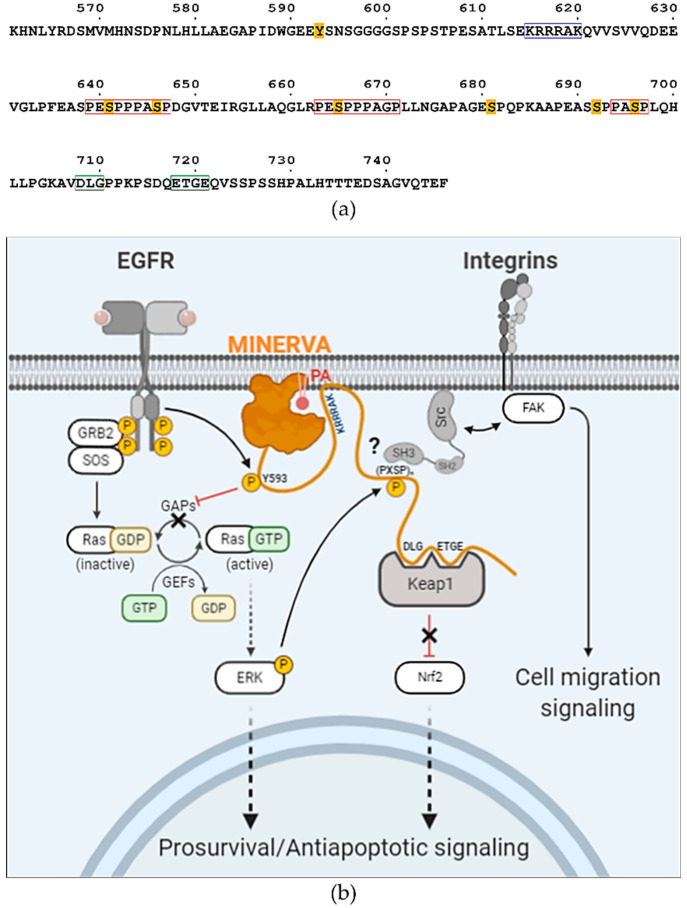
Proposed role of the flexible C-terminal region in functional regulation of MINERVA. (**a**) Amino acid sequence of the C-terminal flexible region (Lys561–Phe574) is shown with known posttranslational modification sites and characteristic sequence motifs. Phosphorylation sites are highlighted in yellow. Short stretch of positively charged residues are in blue frame. Tandem SH3 domain-binding “(PXXPP)P*ψ*XP” motifs are in red frames. Keap1-interacting “DLGX_n_ETGE” motif is in green frame. (**b**) Schematic summarizing known and proposed MINERVA function as a regulator of cell signaling. MINERVA is shown in orange.

Our structure has revealed that the N-terminal myristoylated residue Gly2 is positioned adjacent to the PH domain to constitute a positively charged membrane-interacting face for cooperative action of anchoring MINERVA to lipid membranes (Figure 2d,e). Previous reports, as well as our observation on the localization of MINERVA, showed that the protein is dispersed in cytoplasm as puncta form in exponentially growing HeLa cells, whereas it was recruited to cell–cell contacts whenever the cells became confluent [16]. Contact inhibition is the major factor in switching cellular functions from growth and proliferation to migration [31], and we investigated lipid binding ability of the PH domain on MINERVA to gain insight on the function of the protein. Lipid dot-blot assay indicated that MINERVA^FL^ predominantly binds to PA, whereas MINERVA^ΔC^ showed higher affinity towards PI3P (Figure 3c). We speculate that negatively charged residues (Glu80–Ser82) on the β1–β2 loop at the ligand binding site seem to restrict binding of lipids with multiple phosphate groups, while the side chain of Gln79 only allows binding of lipids with smaller head groups. Although PH domains are best known for their abilities to bind phosphatidylinositols [2], the lipid dot-blot assay results suggest that PA can also be recognized by the PH domain. The difference in binding affinity between MINERVA^FL^ and MINERVA^ΔC^ implies that the C-terminal flexible region contributes to the ligand selectivity of the N-terminal PH domain. Despite the importance of PA in cell signaling [32], domains or motifs responsible for recognizing PA have not been clearly defined. Studies on PA-binding proteins commonly noticed a short stretch of positively charged peptides responsible for the interaction [33]. Sequence analysis of the C-terminal flexible region of MINERVA revealed a similar motif ^615^-KRRRAK-^620^ (Figure 6a), which might be responsible for enhancing ligand specificity of the PH domain on MINERVA^FL^ towards PA. The facts that MINERVA^ΔC^ showed higher affinity against PI3P over PA, and that PI3P was recognized as the innate ligand of MINERVA PH domain alone [29], hint the importance of this positive residue stretch in the C-terminal flexible region for PA recognition. In cells, PI3P is predominantly populated on endosomes, for sorting out protein targets for endocytosis [34]. Moreover, N-terminal myristoylation on Gly2 residue strongly suggests that cytoplasmic fraction of MINERVA is in fact endosome-associated, and the cooperative action of the N-terminal myristic acid and the PH domain is supported by our structure (Figure 2d). We speculate that intracellular localization of MINERVA is regulated by the PH domain, depending on the level of cell confluency [16]. Once cells become confluent, MINERVA would be recycled back to the plasma membrane for activation of cell migratory signaling pathways via, for example, FAK phosphorylation [11].

The “DLGXnETGE” motif within the C-terminal flexible region was pointed out to be responsible for lifting Keap1-mediated Nrf2 degradation [12]. Keap1–Nrf2 is a well-known defense system against cellular oxidative stress which is frequently observed in cancer progression. Nrf2 is ubiquitinated and degraded by interaction with Keap1, a scaffold protein for Cul3-containing E3 ubiquitin ligase, whereas under the conditions of oxidative stress, Nrf2 is disrupted from interaction with Keap1 and then is translocated into the nucleus for transcriptions of antioxidant genes [35,36]. MINERVA competes with Nrf2 for Keap1-binding through the C-terminal region, resulting in Nrf2 activation [12]. Our SPR analysis of MINERVA^FL^ and Keap1 provides direct evidence for physical association between the two proteins with micromolar binding affinity (Figure 5b). Moreover, MINERVA^ΔC^ failed to interact with Keap1, implying the importance of the C-terminal region of MINERVA for cancer progression through Nrf2 mediated regulation (Figure 5a). On the other hand, the effect of phosphoserines on MINERVA function was elusive since the first report of MINERVA as a target of ERK phosphorylation [6]. We noticed that the sequences around these serine residues exhibited a tandem proline-rich consensus motif “(PESPP)PASP”, where underlined serine residues are the targets of phosphorylation and residues in brackets are less conserved (Figure 6a). This motif is reminiscent of a Src specific SH3-domain binding motif “(PXXPP)P*ψ*XP”, where *ψ* and X represent hydrophobic and any amino acid residues, respectively [37,38]. Thus, it would be interesting to further investigate whether the interaction with Src presents a possible role of MINERVA in regulation of cell motility. Overall, the C-terminal flexible region seems to harbor multiple sequence motifs and PTM sites for interaction with other proteins for regulation of signal transduction (Figure 6a,b). We can hypothesize that MINERVA recognizing PA or PI3P by the N-terminal PH domain, as well as EGF-mediated ERK phosphorylation of the C-terminal “(PESPP)PASP” motifs, might allow spatial and temporal regulation of Src activation to promote cell proliferation and migration.

We present the first structure of the globular domain from MINERVA that displays distinct crevices and a cavity shown from surface representations (Figure 3e). We suggest that these characteristic crevices and the cavity provide a binding platform for MINERVA interacting partners. The widths and lengths of the crevices seem adequate for accommodating 20–30 amino acid long loops each. Although a certain region within the C-terminal flexible region may be the inherent occupant of the crevices, we excluded such possibility as crystallization attempts with trypsin-digested MINERVA^FL^ produced mostly the same structure model with no additional electron densities at the crevices. However, it should be noted that the presented structure of MINERVA^ΔC^ might not necessarily represent the in vivo structure since it lacks the C-terminal flexible region harboring post-translational modifications sites and motifs for interaction with other proteins. Binding of interaction partners often elicits conformational changes required for protein function [39]. Interactome analysis indicates that interacting partners of MINERVA includes not only signaling molecules discussed above (EGFR, H-Ras, ERK, and Keap1), but also proteins responsible for endosomal sorting, such as SNX2 and SNX6, and VPS26A, VPS29, and VPS35 (https://thebiogrid.org/122328) [40]. The fact that E-cadherin was suggested as another binding partner of MINERVA hints at the role of MINERVA in cell migration upon confluency [41]. These interacting partners may exploit the crevices and cavity present on the structured domain of MINERVA^ΔC^, since the binding regions of the other signaling molecules are focused on the C-terminal flexible region. Regarding its possible influence over multiple signaling pathways regulating cell growth, proliferation, motility, oxidative stress, and survival and apoptosis, the structural elucidation of MINERVA would present valuable information on cancer progression and therapy.

## 4. Materials and Methods

### 4.1. Cell Lines

All human cell lines were purchased from the American Type Culture Collection (ATCC; Manassas, VA, USA) and were maintained at 5% CO_2_ and 37 °C in Dulbecco’s modified Eagle’s medium (DMEM), Roswell Park Memorial Institute (RPMI) 1640 or Iscove’s Modified Dulbecco’s Medium (IMDM) supplemented with 10% fetal bovine serum (FBS; HyClone, Logan, UT, USA), 100 U/mL penicillin, and 100 μg/mL streptomycin (Gibco, Waltham, MA, USA).

### 4.2. Antibodies and Reagents

The primary antibody against MINERVA (5122) was purchased from Cell Signaling Technology (Danvers, MA, USA) or from NOVUSBIO (NBP1-88783) for western blot or immunofluorescence; those against β-actin (A300-491A) and HA (A190-108A) were purchased from Bethyl Laboratories (Montgomery, TX, USA); those against FLAG M2 (F1804) and FLAG (F7425) were purchased from Sigma-Aldrich (St. Louis, MO, USA) for western blot; those against FLAG (D6W5B) were purchased from Cell Signaling Technology (Danvers, MA, USA) for immunofluorescence. The secondary antibodies, horseradish peroxidase (HRP)-linked anti-rabbit (A120-101P) and anti-mouse (A90-116P), were purchased from Bethyl Laboratories (Montgomery, TX, USA). Lipofectamine 2000 (11668019) and RNAiMAX (13778150) were purchased from Thermo Fisher Scientific (Waltham, MA, USA).

### 4.3. DNA Construct and siRNA

For the construction of the FLAG-MINERVA, a PCR-amplified DNA fragment encoding MINERVA was inserted between the *NotI* and *XbaI* sites of the pcDNA3 or pcDNA3.1 vector. The deletion mutants of the FLAG-MINERVA (MINERVA^ΔC^; residues Met1–Ser574 and MINERVA^ΔN^; residues Thr307–Phe746) were constructed with *NotI* and *XbaI* sites of the pcDNA. siRNA targeting the respective genes of interest and negative control siRNA (non-targeting pool) were purchased from Genolution Inc. (Seoul, Korea). The following siRNA sequences were used for the indicated target genes; siMINERVA_#1 5′-UCACGGACAUGAACCUGAACGUCAU-3′ and siMINERVA_#2 5′-CAGUAUGGCGUGGCUCUCUUCAACA-3′.

### 4.4. Immunoprecipitation

MINERVA and its truncated forms were tagged with a FLAG epitope and Keap1 was tagged with a human influenza HA epitope. Each combination of the proteins was co-expressed in HEK293T cells. HEK293T cells were rinsed in ice-cold PBS and lysed in lysis buffer composed of 1% NP-40, 20 mM Tris-HCl (pH 7.5), 150 mM NaCl, 10% glycerol, 2 mM ethylenediaminetetraacetic acid (EDTA), 10 mM NaF, 1 mM Na_3_O_4_V, and 0.2 mM phenylmethylsulfonyl fluoride (PMSF), including a protease inhibitor cocktail (11836153001; Roche Applied Bioscience, Penzberg, Germany) and 1% phosphatase inhibitor cocktail (Sigma-Aldrich, St. Louis, MO, USA). Next, 0.5 mg lysates for co-immunoprecipitation in cells overexpressing the above proteins were incubated with 2 μg primary antibodies for anti-FLAG M2 (Sigma-Aldrich, St. Louis, MO, USA) or anti-HA antibody (Bethyl Laboratories, Montgomery, TX, USA), or rabbit IgG (Sigma-Aldrich, St. Louis, MO, USA) at 4 °C with overnight shaking after adding 50 μL protein A agarose beads (GenDEPOT, Katy, TX, USA). Immunoprecipitates were washed three times with wash buffer and then eluted by boiling in sodium dodecyl sulfate (SDS) sample buffer with β-mercaptoethanol for 5 min. Then, the immunoprecipitate complex was immunoblotted with the indicated antibodies.

### 4.5. Cell Proliferation and Viability Assays

siRNA-transfected cells were seeded at 4 × 10^3^ per well in a 96-well plate, which was placed in the IncuCyte ZOOM™ system (Essen BioScience, Ann Arbor, MI, USA). After incubation for the indicated times live-cell images were obtained using a 10× objective lens (four images per well) within the instrument, and cell density was analyzed using IncuCyte ZOOM 2016B software.

Cell viability was determined by annexin V and PI staining following standard protocols at the indicated time periods (556547, BD Biosciences, San Jose, CA, USA). Cells negative for both annexin V and PI were considered live cells. The proportion of dead cells was measured based on the number of annexin V and PI single and both-stained cells. The fluorescence of stained cells was detected using the LSRFortessa fluorescence activated cell sorting (FACS) analyzer (BD Biosciences).

### 4.6. Western Blotting

Cells were harvested in ice-cold radioimmunoprecipitation assay (RIPA) lysis buffer (50 mM Tris-HCl (pH 7.4), 150 mM NaCl, 1% NP-40, 0.5% Na-deoxycholate, 0.1% SDS, and 1 mM EDTA) containing protease inhibitor cocktail (Roche Applied Bioscience, Penzberg, Germany) and phosphatase inhibitor (Sigma-Aldrich, St. Louis, MO, USA). Soluble lysate fractions were isolated by centrifugation at 20,000× *g*, for 20 min at 4 °C and quantified using the Pierce bicinchoninic acid (BCA) Protein Assay kit (Thermo Fisher Scientific, Waltham, MA, USA). Samples were resolved by sodium dodecyl sulfate-polyacrylamide gel electrophoresis (SDS-PAGE) using equal concentrations of protein and transferred to PVDF membranes. The membranes were blocked with 5% skim milk and then probed with the indicated primary and secondary antibodies following standard protocols. Immunoblotted proteins were quantified using ImageJ software (NIH, Bethesda, MD, USA). Data are expressed as the mean ± standard deviation (SD) or standard error of the mean (SEM) which are from at least three independent experiments. Statistical significance was calculated using Student’s *t*-test in GraphPad Prism. A value of *p* < 0.05 was considered statistically significant (* *p*  < 0.05; ** *p*  < 0.01).

### 4.7. Immunofluorescence Analysis

To determine the localization of MINERVA, immunostaining against MINERVA was performed using Hela cells. After they were plated onto glass coverslips in complete medium for 18 h, cells were incubated in control (3 × FLAG only) and MINERVA domain construct mixture treatment for 48 h. For immunostaining, cells were rinsed with ice-cold PBS, fixed with 3.7% formaldehyde for 20 min, and permeabilized with 0.3% Triton X-100 in PBS (PBS-T) for 5 min. After rinsing with PBS-T, cells were blocked with blocking buffer (5% BSA in PBS-T) for 1 h, incubated with primary antibodies against MINERVA or FLAG in blocking buffer for 16 h in 4 °C, washed three times with PBS-T and then incubated with secondary antibodies conjugated with fluorescent probe in PBS-T for 1 h at room temperature. After three PBS-T washes, cells were stained with DAPI (4′,6-diamidino-2-phenylindole) for 10 min and mounted on microscope slides. A confocal fluorescence microscope (Zeiss LSM 780, Oberkochen, Germany) was used for imaging and analysis.

### 4.8. Cloning, Expression, and Purification of Recombinant MINERVA Constructs for Structure Determination and Affinity Assay

The full length MINERVA^FL^ (residues Met1–Phe746) and C-terminally truncated human MINERVA^ΔC^ (residues Met1–Ser574) were amplified using PCR and cloned into pET-28a(+) and pET-21a(+) vectors (Novagen, Burlington, MA, USA) to each contain an N-terminal or a C-terminal His_6_-tag, respectively. The plasmids containing the MINERVA constructs were each transformed into Rosetta 2(DE3)pLysS or BL21-CodonPlus (DE3)-RIPL *Escherichia coli* strain, respectively.

The transformed cells were grown for 16 h in Luria Broth media without induction to be used as seeds for the scale-up culture. The uninduced *E. coli* seeds containing MINERVA plasmids were inoculated into a scale-up culture media at a ratio of 1:50. The cells in scale-up culture were grown in Luria Broth media and induced with 0.5 mM isopropyl β-D-1-thiogalactopyranoside, followed by further incubation at 20 °C for 16 h. Harvested cells were lysed by a cell sonicator (SONICS) in a lysis buffer containing 20 mM Tris-HCl (pH 7.5), 500 mM NaCl, 35 mM imidazole, and 1 mM PMSF. Cell debris was removed by centrifugation at 35,000× *g* for 50 min at 4 °C, then the supernatant was filtered with 0.22 μm filter to remove cell debris and any aggregated proteins. The filtered supernatant was applied onto a HiTrap Chelating HP column (GE Healthcare, Chicago, IL, USA) equilibrated with the lysis buffer for affinity chromatography. Unbound or weakly bound proteins were removed from the HiTrap Chelating HP column with six column volume elutions of the washing buffer containing 20 mM Tris-HCl (pH 7.5), 500 mM NaCl, and 50 mM imidazole. The retained proteins were eluted with a gradually increasing addition of a buffer containing 20 mM Tris-HCl (pH 7.5), 500 mM NaCl, and 500 mM imidazole. The eluted protein samples were further purified by size-exclusion chromatography with HiLoad 16/600 Superdex200 pg column (GE Healthcare, Chicago, IL, USA) equilibrated with a buffer containing 20 mM Tris-HCl (pH 8.0) and 200 mM NaCl. The purification steps for SeMet-substituted MINERVA^ΔC^ were as above except that the buffer for the final size exclusion chromatography additionally contained 1 mM tris(2-carboxyethyl)phosphine (TCEP).

### 4.9. Cloning, Expression, and Purification of Keap1^321–609^ for Affinity Assay

N- and C-terminally truncated Keap1 protein (residues Ala321–Thr609) containing the Kelch domain was amplified using PCR and cloned into the pET-28a(+) vector to contain an N-terminal His_6_-tag. The plasmid containing Keap1^321–609^ was transformed into Rosetta 2(DE3)pLysS *E*. *coli* strain. The purification steps for Keap1^321–609^ were as above except that the buffer for the final size exclusion chromatography contained 20 mM Tris-HCl (pH 7.5) and 5 mM dithiothreitol (DTT).

### 4.10. Limited Trypsin Digestion of MINERVA Proteins

Native and SeMet-substituted MINERVA^ΔC^ were subjected to digestion by trypsin (Sigma-Aldrich, St. Louis, MO, USA) by incubating purified MINERVA^ΔC^ at a 5000:1 trypsin ratio at 4 °C for 20 h prior to crystallization in order to improve diffraction quality of crystals. Digested proteins were further purified by size-exclusion chromatography with HiLoad 16/600 Superdex200 pg column equilibrated with a buffer containing 20 mM Tris-HCl (pH 8.0) and 200 mM NaCl. The purified trypsin-treated MINERVA^ΔC^ proteins were concentrated to 4.8 mg mL^−1^ for crystallization.

### 4.11. Crystllization, Data Collection, and Structure Determination

Crystals of the trypsin-treated SeMet-substituted MINERVA^ΔC^ were grown at 22 °C by the hanging drop vapor diffusion method by mixing equal volumes of the protein at 4.8 mg mL^−1^ and a crystallization solution containing 0.2 M sodium tartrate and 20% (*w/v*) polyethylene glycol (PEG) 3350. Crystals of the trypsin treated native MINERVA^ΔC^ were grown at 22 °C by the same methods with a crystallization solution containing 0.2 M lithium acetate and 20% (*w/v*) PEG 3350. Crystals of diffraction quality were cryoprotected with the reservoir solution containing additional 25% (*v/v*) glycerol and flash-cooled in a nitrogen gas stream at 100 K. Diffraction data from the SeMet-substituted and the native crystals were collected up to resolutions of 2.39 Å and 1.65 Å, respectively. Raw X-ray diffraction data were indexed and scaled using the HKL-2000 program suite or the XDS program package [42,43]. Single-wavelength anomalous diffraction (SAD) phases of the SeMet-substituted MINERVA^ΔC^ were initially calculated with AUTOSOL in the PHENIX software suite [44] and further improved by the automatic model building program RESOLVE [45], resulting in an initial model. The initial model was further refined to the final model using iterative cycles of model building with Coot [46] and subsequent refinement with Refmac5 in the CCP4 suite [47] and phenix.refine [48]. The crystal structure of the native MINERVA^ΔC^ was determined by molecular replacement with the MolRep program [49], using the refined structure of SeMet-substituted MINERVA^ΔC^ as a phasing model. Validation of crystal structures was implemented with MolProbity [50] and the Research Collaboratory for Structural Bioinformatics (RCSB) Protein Data Bank (PDB) validation server. Statistics for the data collection and refinement are summarized in Table 1. The coordinate and structure factor of the native MINERVA^ΔC^ have been deposited in the PDB (http://www.rcsb.org) under accession code 7CTP.

### 4.12. Size-Exclusion Chromatography with Multi-Angle Light Scattering (SEC–MALS)

The oligomeric states of the recombinant MINERVA^FL^ and MINERVA^ΔC^ proteins were assessed by SEC–MALS experiments using an ÄKTA fast protein liquid chromatography (FPLC) system (GE Healthcare, Chicago, IL, USA) connected to a Wyatt DAWN Heleos II MALS instrument and a Wyatt Optilab T-rEX differential refractometer. A Superdex 200 Increase 10/300 GL gel-filtration column (GE Healthcare, Chicago, IL, USA), which was pre-equilibrated with buffer A containing 20 mM Tris-HCl (pH 8.0) and 200 mM NaCl, was normalized using ovalbumin. The proteins (2 mg) were injected at a flow rate of 0.5 mL min^−1^. Data were analyzed using the Zimm model for fitting static light-scattering data and were graphed using Easy Analytic Software Inc. (EASI) graph with a UV peak in the ASTRA 6 software (Wyatt, Santa Barbara, CA, USA).

### 4.13. In Silico Docking Experiment

Induced fit docking (IFD) experiment (Schrödinger Suite Induced Fit Docking protocol; Glide version 7.4, Prime version 4.7, Schrödinger, LLC, New York, NY, USA) was implemented in order to predict the binding mode of lipids to the PH domain of MINERVA. The MINERVA^ΔC^ structure was prepared for docking calculations using the Protein Preparation Wizard implemented in Maestro (Schrödinger, LLC, New York, NY, USA). The structure of MINERVA^ΔC^ was built in Maestro 8.5, and the possible conformations of the lipids were generated using LigPrep (version 4.1, Schrödinger, LLC, New York, NY, USA). The head groups of lipids were docked onto MINERVA^ΔC^ using the following steps. (i) The receptor grid of MINERVA^ΔC^ was defined as an enclosing box at the centroid of the key amino acid residues in the binding pocket. (ii) In the initial Glide docking stage, a potential docking with van der Waals radius scaling of 0.5 for the protein and ligand was performed retaining a maximum number of 20 poses per ligand. (iii) Residues within 5.0 Å of ligand poses were kept free to move in the Prime refinement step, and the side chains were further optimized. (iv) Poses within 30 kcal mol^−1^ of the energy cutoff in the previous step were re-docked using Glide XP. (v) The binding free energy (ΔG_bind_) for each output pose was computed using Prime-MMGBSA (version 3.0).

### 4.14. Lipid Dot Blot Assay

The PIP strips (Echelon Biosciences, Salt Lake City, UT, USA) were blocked with phosphate-buffered saline containing 0.5% (*v/v*) Tween-20 (Amersham Biosciences, UK) and 3% (*w/v*) bovine serum albumin (BSA; Bovogen, East Keilor, Australia) for 1 h and incubated with MINERVA^FL^ or MINERVA^ΔC^ for 1 h. MINERVA^FL^ bound strips were treated with α-MINERVA rabbit antibody (5122; Cell Signaling Technology, Danvers, MA, USA) for 1 h then with HRP-conjugated α-rabbit IgG goat antibody (A120-101P; Bethyl Laboratories, Montgomery, TX, USA) for 1 h. MINERVA^ΔC^ bound strips were treated with HRP-conjugated α-His_6_-tag antibody (Santa Cruz Biotechnology, Dallas, TX, USA). The strips were washed with the blocking buffer for 5 min three times between each step. The strips were visualized with an ECL kit (Amersham Biosciences, UK).

### 4.15. Surface Plasmone Resonance (SPR) Analysis of MINERVA

SPR experiments for the interaction of MINERVA^FL^ with Keap1^321–609^ were carried out in a Biacore T200 system (GE Healthcare, Chicago, IL, USA) using a buffer comprising 10 mM HEPES and 150 mM NaCl at pH 7.4. MINERVA^FL^ in 10 mM sodium acetate at pH 5.0 was immobilized by the amine coupling method on a CM5 sensor chip according to the manufacturer’s protocol (GE Healthcare, Chicago, IL, USA). A reference flow cell was equally treated but without protein as a control and the response by the control was subtracted from each sample. Keap1^321–609^ at concentrations of 78.1, 156, 313, 625, 1250, 2500, 5000, and 10,000 nM were injected over the MINERVA^FL^ chip at a rate of 30 μL min^−1^. Biacore T200 evaluation software was used to estimate the affinity and kinetics fittings using the steady state affinity model and 1:1 binding model, respectively, to calculate the kinetics data. Average and standard deviation were calculated from two separate kinetics’ data measured.

## Figures and Tables

**Table 1:** Data collection and refinement statistics for MINERVA^ΔC^ crystal structure.

PDB Entry	7CTP
Diffraction source	SPring-8 BL44XU
Wavelength (Å)	1.0000
Temperature (K)	100
Space group	*P2*_1_ *2*_1_ *2*_1_
a, b, c (Å)	55.559, 56.283, 201.01
α, β, γ (°)	90, 90, 90
Resolution range (Å) *^a^*	49.11–1.80 (1.86–1.80)
Total No. of reflections *^a^*	443,604 (43,898)
No. of unique reflections *^a^*	59,470 (5838)
Completeness (%) *^a^*	99.98 (99.98)
Redundancy *^a^*	7.5 (7.5)
〈I/σ(I)〉 *^a^*	18.24 (2.24)
*R*_meas_ *^a^*	0.063 (0.825)
*R*_pim_ *^a^*	0.023 (0.299)
*CC_1/2_* *^a^*	0.999 (0.799)
Overall B factor from Wilson plot (Å^2^)	30.86
No. of reflections, working set *^a^*	59,467 (5838)
No. of reflections, test set *^a^*	2925 (256)
Final *R*_work_ *^b^*	0.2038
Final *R*_free_ *^b^*	0.2355
No. of non-H atoms	4806
Protein	4519
Glycerol	18
Water	269
Root-mean-square deviations	
Bonds (Å)	0.007
Angles (°)	1.08
Average B factors (Å^2^)	37.41
Protein	37.33
Glycerol	45.58
Water	37.33
Ramachandran plot *^c^*	
Favored (%)	99.45
Allowed (%)	0.55

*^a^* Values in parentheses refer to the highest resolution shell. *^b^ R*_work_ = Σ||*F*_obs_| − |*F*_calc_||/Σ|*F*_obs_|, where *R*_free_ is calculated for a randomly chosen 5% of reflections, which were not used for structure refinement and *R*_work_ is calculated for the remaining reflections. *^c^* Values obtained using MolProbity.

## Supplementary Materials

The following are available online at https://www.mdpi.com/1422-0067/21/21/8186/s1.

## Abbreviations

MINERVA	Melanoma invasion by ERK
PH domain	Pleckstrin homology domain
PTM	Posttranslational modification
SAD	Single-wavelength anomalous diffraction
MRASU	Molecular replacementAsymmetric unit
PA	Phosphatidic acid
PI3P	Phosphatidylinositol (3)-phosphate
*k_a_*	Association rate constant
*k_d_*	Dissociation rate constant
*K_D_*	Equilibrium dissociation constant
