# Supplementary material for: Structural Insight on Functional Regulation of Human MINERVA Protein
Source: Int J Mol Sci. 2020 Oct 31;21(21):8186. doi: 10.3390/ijms21218186 (PMC7663100; doi:10.3390/ijms21218186)
Supplement: Supplementary file 1 [file ijms-21-08186-s001.pdf]

International Journal of *Molecular Sciences*

Article

Structural Insight on Functional Regulation of Human MINERVA Protein

Hyunggu Hahn [1,2], Dong-Eun Lee [3], Dong Man Jang [2], Heesun Cheong [1,3,*], Byung Woo Han [2,*], and Hyoun Sook Kim [1,*]

[1] Research Institute, National Cancer Center, Goyang-si, 10408 Gyeonggi-do, Republic of Korea.
[2] Research Institute of Pharmaceutical Sciences, College of Pharmacy, Seoul National University, 08826 Seoul, Republic of Korea.
[3] Graduate School of Cancer Science & Policy, National Cancer Center, Goyang-si, 10408 Gyeonggi-do, Republic of Korea.

* Correspondences: heesunch@ncc.re.kr; Tel.:+82-31-920-2272 (H.C.); bwhan@snu.ac.kr; Tel.:+82-2-880-7898 (B.W.H.); hskim@ncc.re.kr; Tel.:+82-31-920-2275 (H.S.K.)

Received: date; Accepted: date; Published: date

Supplementary Data

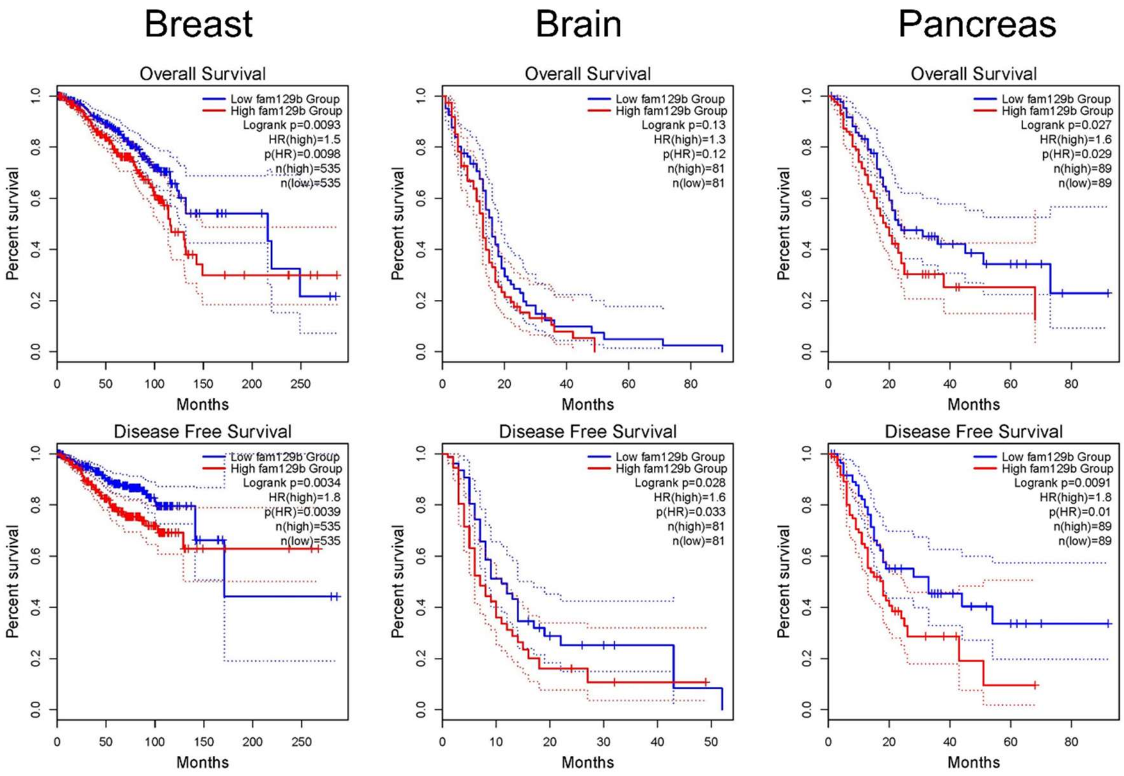

Figure S1. K-M plots of overall survival rate of breast invasive carcinoma (BRCA), glioblastoma multiforme (GBM), pancreatic adenocarcinoma (PAAD) patients expressing MINERVA (FAM129B). Patients expressing high levels of MINERVA show lower survival rate.

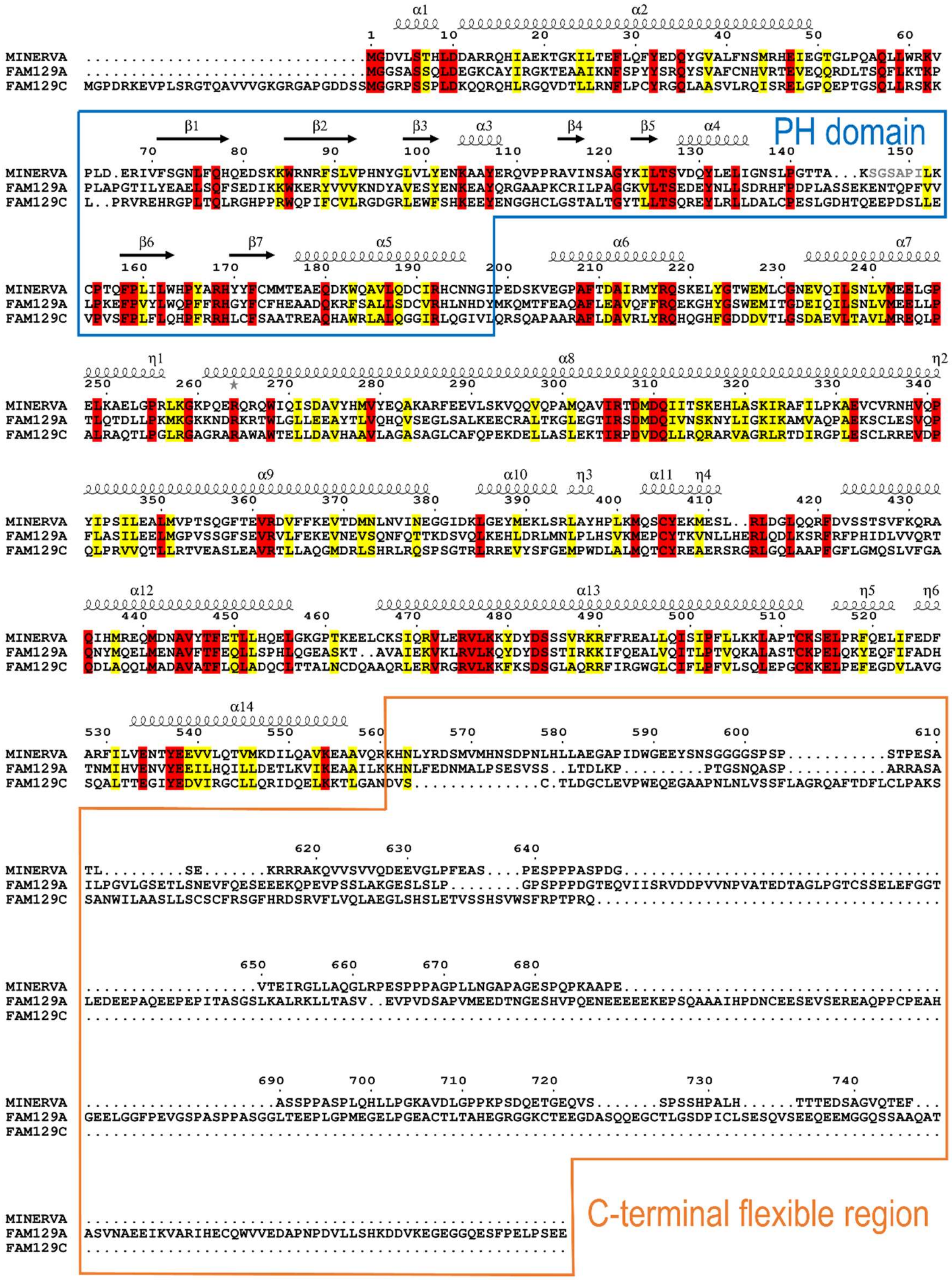

Figure S2. Multiple sequence alignment of FAM129 family members MINERVA (FAM129B), FAM129A, and FAM129C. Regions corresponding to the structured domain of MINERVA (Gly2–Arg560) are well-conserved among the family members. Identical and similar residues are highlighted in red and yellow, respectively. PH domain and the C-terminal flexible region are indicated by blue and orange boxes, respectively. The C-terminal flexible regions vary greatly in length and sequence.

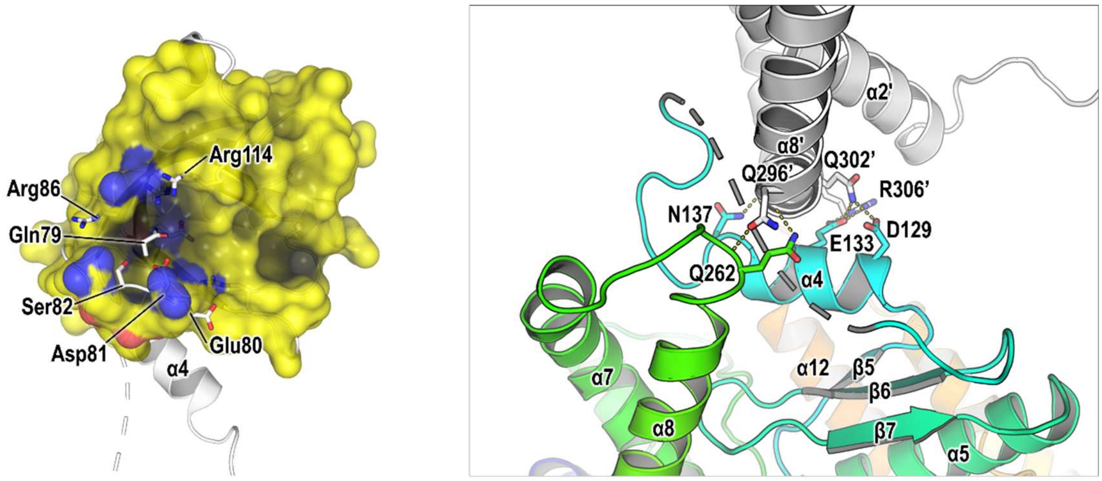

(a) (b)

Figure S3. PH domain of MINERVA. (a) Superposition of the PH domains from MINERVA (white cartoon and stick representations) and Pleckstrin (yellow surface representation) in the same view as Figure 3b, left. Side chains of residues that give rise to ligand specificity to MINERVA PH domain are shown in stick representations and labeled (Gln79, Glu80, Asp81, and Ser82). Pleckstrin possesses Gly255, His256, Arg257, and Arg258 on the corresponding positions, that allow larger binding pocket as displayed by the surface representation. (b) Intermolecular interaction between two adjacent molecules of MINERVA establish multiple hydrogen bonds, indicated with yellow dashed lines. The PH domain of a MINERVA molecule is shown as a cartoon representation in cyan-to-green color while helix $\alpha 8'$ of the adjacent MINERVA is in white. The unmodeled region (Ser146–Ile151) are indicated with gray dashed ribbon.

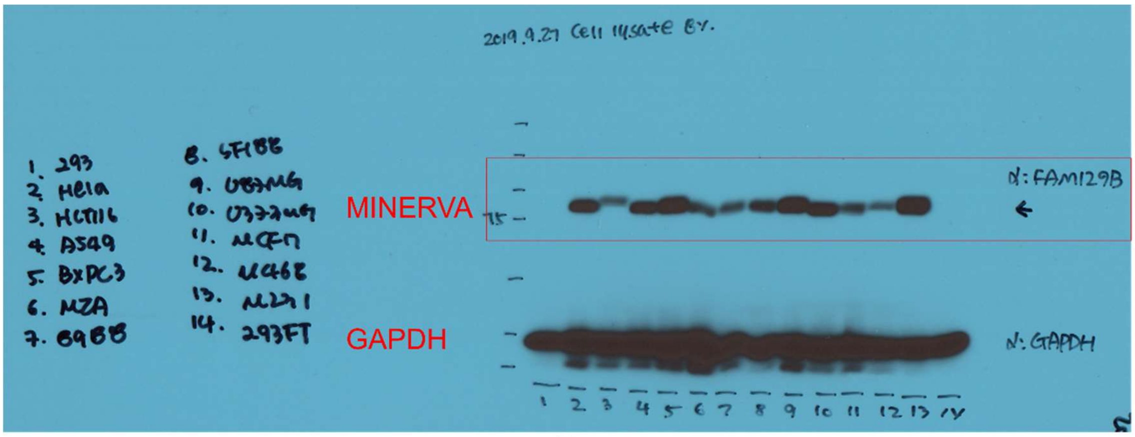

(a)

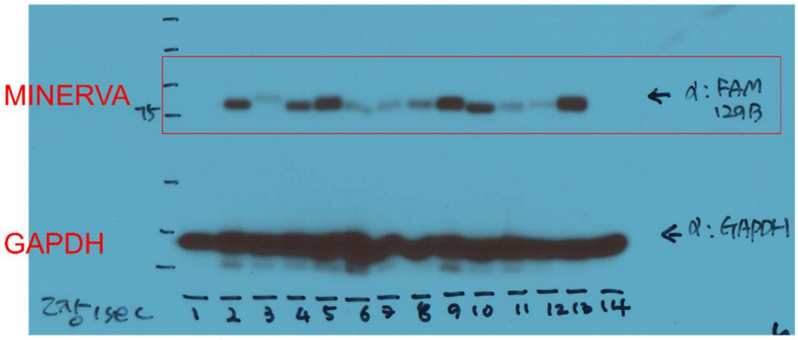

(b)

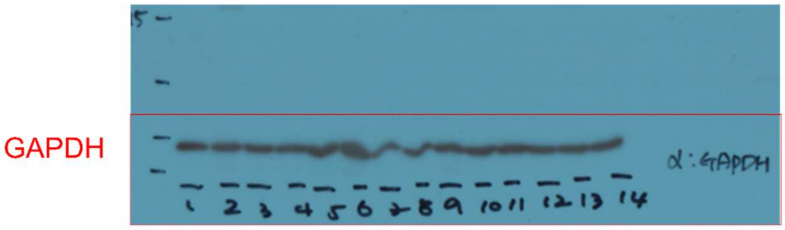

(c)

Figure S4. Full Western blots for Figure 1a from same membrane. (a) Long exposure. (b) Medium exposure. (c) Short exposure for GAPDH.

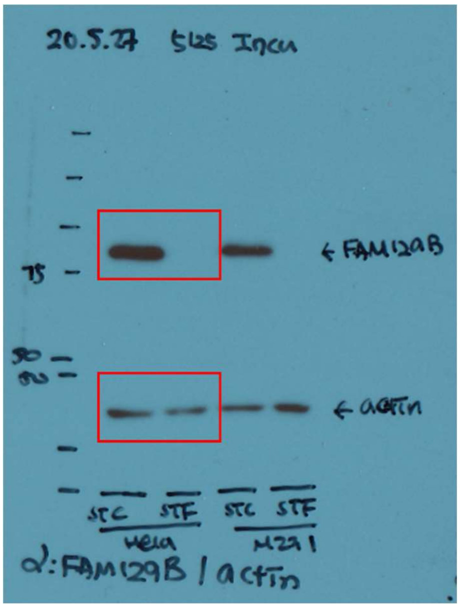

Figure S5. Full Western blot for Figure 1b.

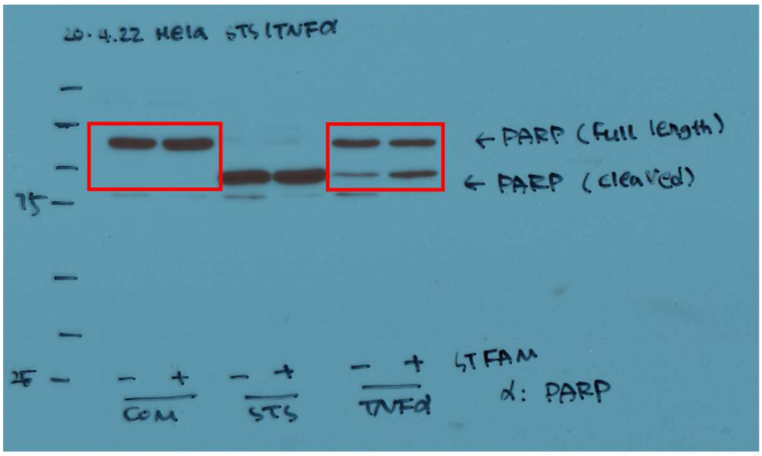

(a)

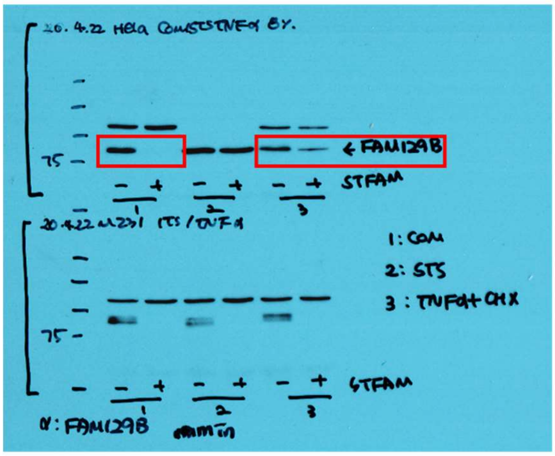

(b)

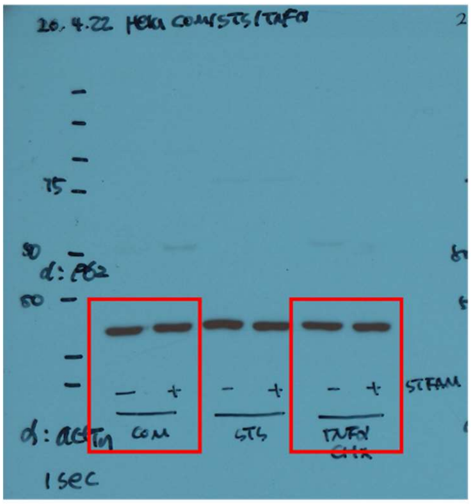

(c)

Figure S6. Full Western blots for Figure 1d. Sequential blots against (a) anti-PARP, (b) anti-MINERVA (FAM129B), and (c) anti-Actin (loading control).

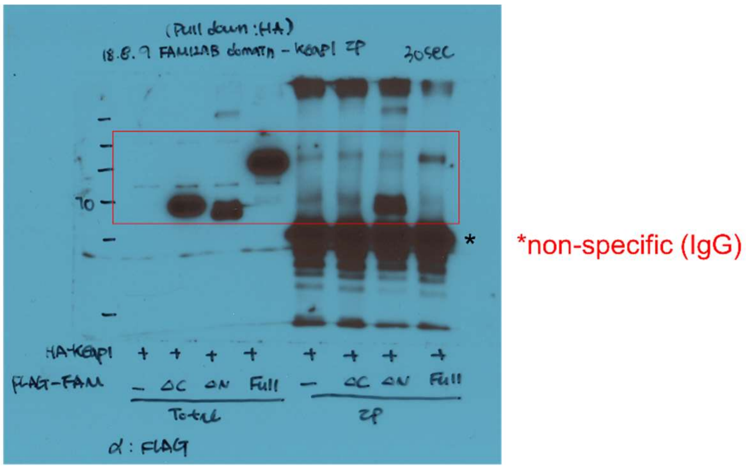

(a)

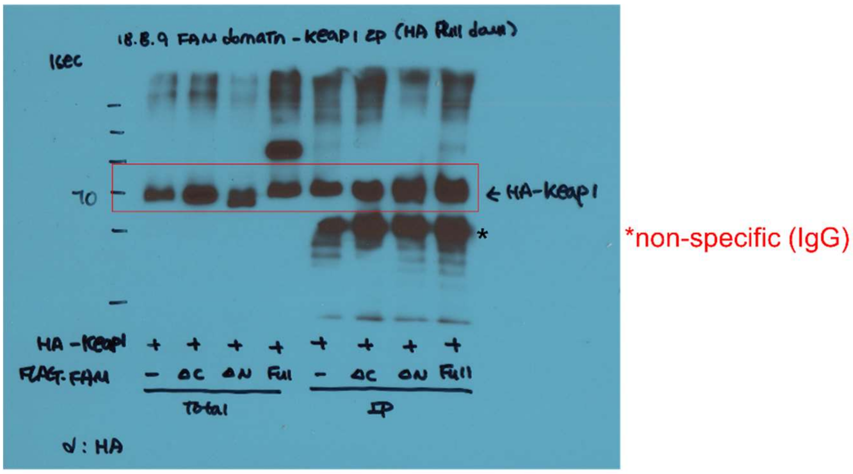

(b)

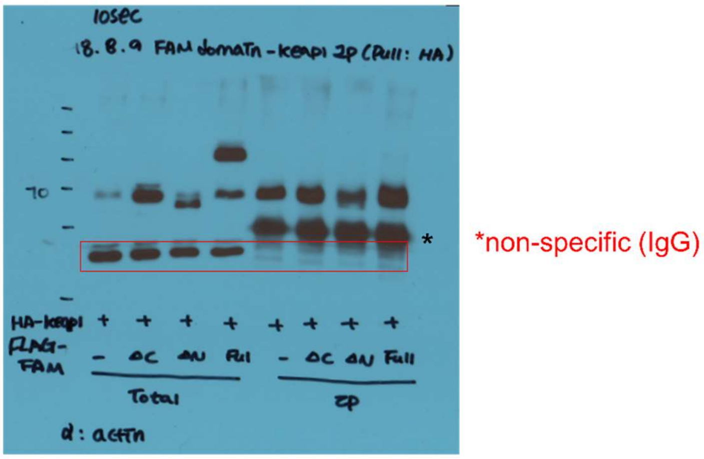

(c)

Figure S7. Full Western blots for Figure 5a. Sequential blots on same membrane against (a) anti-FLAG, (b) anti-HA, and (c) anti-Actin (loading control).
